# Tricuspid regurgitation: remembering ‘the forgotten valve’

**DOI:** 10.1007/s12471-022-01714-7

**Published:** 2022-08-03

**Authors:** R. J. de Winter

**Affiliations:** Department of Cardiology, Amsterdam University Medical Centers, Amsterdam, The Netherlands

The tricuspid valve, separating the right atrium from the right ventricle, is a complex structure with its fibrous saddle-shaped annulus, three leaflets, two papillary muscles and chordae tendineae, and is sometimes referred to as ‘the forgotten valve’. In recent years, the tricuspid valve has been rediscovered and tricuspid regurgitation (TR) is now recognised as a not uncommon cause of dyspnoea on exertion and progressive fluid retention. It is estimated that clinically significant TR is present in 4% of subjects over 75 years of age [1, 2]. Primary causes of TR occur in 25% of cases and include rheumatic disease, endocarditis, carcinoid disease, Ebstein anomaly, trauma and iatrogenic causes, e.g. pacemaker leads. Secondary causes account for the remaining 75% and include TR due to pulmonary hypertension as a consequence of left heart disease (left ventricular dysfunction, valve disease), pulmonary hypertension in the setting of lung disease, chronic pulmonary embolism or right ventricular dysfunction, e.g. due to ischaemia/infarction. TR results in volume overload of the right ventricle and annular dilatation with worsening of regurgitation, resulting in atrial enlargement with atrial fibrillation. This vicious circle leads to progressive heart failure and eventually significant morbidity.

Treatment of TR includes intensive use of diuretics, in cases of atrial fibrillation rate or rhythm control, precapillary pulmonary hypertension medication and addressing left heart disease with coronary revascularisation and treatment of valve disease where appropriate. Surgical treatment for isolated TR has been associated with high mortality (8.8–9.7%) [3, 4], although this may partly be related to the timing of surgery, with better results with less sick, younger patients [5, 6].

Transcatheter treatment options for severe TR have recently been shown to hold great promise, with most experience being reported with tricuspid transcatheter edge-to-edge repair (T-TEER). Other transcatheter techniques are being explored (annuloplasty, e.g. Cardioband, Millipede; valve implantation, e.g. Intripid, Cardiovalve, bicaval valves, e.g. TricValve, Tricento) but fall outside the scope of this editorial. Several considerations in the application of edge-to-edge repair deserve careful evaluation in planning and executing such procedures. It has become apparent that there is an optimal time window for patient selection, with patients having progressive symptoms but presenting not too late in the course of the disease. By the time severe right ventricular dysfunction occurs, irreversible liver cirrhosis, severely impaired renal function or irreversible precapillary and postcapillary pulmonary hypertension, transcatheter treatment will yield disappointing results. In addition, assessment of tricuspid valve anatomy is crucial because large variation in morphology exists with additional leaflets or leaflet scallops in the septal, posterior and anterior part of the valve [7]. Moreover, due to disease advancement and progressive annular dilatation, the leaflet coaptation gap will become too large and edge-to-edge repair becomes difficult. Imaging with transoesophageal echocardiography is the key to success and requires the presence of adequate echo windows in order to assess the anatomy, the origin of the regurgitant jet(s), the optimal positioning of the clips and the efficacy of the grasping of the leaflets. Obviously, this requires experience and great skills of the interventional imaging cardiologist and seamless collaboration among the interventional operators.

In this issue of the *Netherlands Heart Journal*, Krikken and colleagues from the University Medical Centre in Groningen provide an excellent and concise overview of the current state of transcatheter treatment of TR, covering patient selection, imaging, and the specifics of the procedure [8]. With regard to imaging, they recommend a transoesophageal echocardiogram prior to the procedure to confirm adequate echo windows. They also report their initial experience with the TriClip T‑TEER device (Abbott) in their first 17 patients. They demonstrate that the procedure was safe and resulted in a reduction to TR grade 2 or less in 70% of patients which was accompanied by an improvement in symptoms. There is a learning curve, but this is modest in centres with ample experience with MitraClip (Abbott). The authors summarise that more studies are needed to document the clinical value of T‑TEER in patients with severe TR and emphasise that we should take action to be able to offer this treatment to our patients in the Netherlands.

Last year, the 1‑year results of the TRILUMINATE trial were published [9]. This was an international, prospective, single-arm, multicentre study (*n* = 85) investigating safety and performance of the TriClip tricuspid valve repair system in patients with moderate or more severe TR. At 1 year, TR was reduced to moderate or less severe in 71% of subjects compared with 8% at baseline. Patients experienced significant clinical improvements in New York Heart Association functional class I/II (31% to 83%) 6‑min walk test (272.3 ± 15.6 to 303.2 ± 15.6 m) and Kansas City Cardiomyopathy Questionnaire score (improvement of 20 ± 2.61 points). Significant reverse right ventricular remodelling was observed in terms of size and function. The overall major adverse event rate and all-cause mortality were both 7.1% at 1 year. Several randomised studies comparing T‑TEER with optimal medical therapy are underway (TRILUMINATE pivotal trial (NCT03904147, *n* = 700), CLASP II TR trial (NCT04097145, *n* = 825), TRI-FR trial (NCT04646811, *n* = 300)) and more results are expected in the near future. European guidelines on the management of valvular heart disease include a class IIb recommendation for transcatheter treatment of symptomatic secondary severe TR in inoperable patients at a heart valve centre with experience in the treatment of tricuspid valve disease [10]. At present, T‑TEER is not reimbursed in the Netherlands, limiting widespread application. By June 2022, approximately 80 procedures with TriClip had been performed in the Netherlands. The multicentre TRACE-NL study, comparing T‑TEER with optimal medical therapy, will be started soon with Dr. Martin Swaans as lead investigator. Endpoints will be major cardiovascular events, hospitalisation and quality of life at 12 months, and the analysis will include safety and cost-effectiveness. Let us hope that results will become available soon and let us also not forget either the valve or the patients who may benefit from this important and innovative transcatheter treatment.
